# Higher Serum Dipeptidyl Peptidase-4 Activity in Newly Diagnosed Young-Onset Type 2 Diabetes Mellitus

**DOI:** 10.7759/cureus.70968

**Published:** 2024-10-06

**Authors:** Tahseen Mahmood, Hadisur Rahman, Sharmin Jahan, Nusrat Sultana, Mashfiqul Hasan, Md. Imrul Hasan, Md. Abu Shehab, Md Fariduddin, Md. Salimullah, M A Hasanat

**Affiliations:** 1 Endocrinology, Bangabandhu Sheikh Mujib Medical University, Dhaka, BGD; 2 Molecular Biotechnology, National Institute of Biotechnology, Dhaka, BGD

**Keywords:** bangladesh, dm, dpp4 activity, newly diagnosed, young t2dm

## Abstract

Background

Dipeptidyl peptidase-4 (DPP4) enzyme inhibition is a therapeutic modality for type 2 diabetes mellitus (T2DM). However, its activity is insufficiently explored yet in youth-onset T2DM.

Objective

The objective of this study is to measure serum DPP4 enzyme activities in youth-onset T2DM and compare them with those in patients with normal glucose tolerance (NGT).

Methods

This cross-sectional study comprised 29 young participants with newly diagnosed untreated T2DM (age 24.5±4.5 years, range 13-30 years, 51.7% female) and an equal number of young participants with NGT (age 26.5±2.9 years, 48.3% female) screened by a two-sample 75gm oral glucose tolerance test. The relevant history of each participant was obtained, and the waist circumference, waist-hip ratio, blood pressure, and body mass index (BMI) were measured according to the standard procedure and recorded in the data collection sheet. Plasma glucose was measured using the glucose oxidase method, and serum DPP4 activity was measured using the colorimetry method.

Results

Participants with DM exhibited significantly higher serum DPP4 activity than NGT (DM vs. NGT: 481.4±70.4 pmol/min. vs. 420.3±49.2 pmol/min, mean±SD; p<0.001). A multivariate linear regression model adjusted for age, sex, BMI, and hypertension, found DM as an independent predictor for DPP4 activity (β=0.63.5, 95% CI 28.7-98.4, p=0.022).

Conclusion

DPP4 activity was signiﬁcantly elevated in young participants with newly diagnosed T2DM. There might be a pathophysiological implication of DPP4 activity in young-onset T2DM.

## Introduction

The epidemiologic evidence accumulated over the past few decades has revealed that 4 out of 5 patients with diabetes mellitus (DM) live in low- and middle-income countries, and Asians develop diabetes at younger ages with a risk of developing complications in early adulthood. As such, DM has become a burning issue in youth and adolescents [1]. Several factors among Asians have been implicated in its predisposition, including high prevalence of abdominal or central adiposity, high rates of gestational diabetes in combination with in-utero exposure to poor nutrition, childhood obesity, and over-nutrition in later life in addition to genetics. However, missing links interplaying within the complex pathways of diabetes pathogenesis are still being pursued. Furthermore, the observation that young Asians develop diabetes at lower degrees of obesity and at much higher rates given the same amount of weight gain, much interest has emerged to look upon the pathogenesis of diabetes from newer perspectives [2]. Moreover, the increasing diagnosis of type 1 diabetes mellitus (T1DM) in adults and the rising incidence of type 2 diabetes mellitus (T2DM) in childhood and young people have masked the classical perception of these two main types of diabetes as diseases of childhood and adulthood, respectively [3,4]. Regardless of the primary types of diabetes, the central role of inflammation and immune dysregulation is common in the pathogenesis of DM [5]. The possibility of the presence of factor(s) that could influence both the major types of DM situated at the crossroads could affect the understanding of the pathogenesis and therapeutics of DM from a newer perspective.

The enzyme dipeptidyl peptidase-4 (DPP4) inhibition has emerged as one of the novel targets via specific classical and non-classical mechanisms among the diverse therapeutic aspects of T2DM owing to its ability to degrade incretins glucagon-like peptide-1 (GLP-1) and glucose-dependent insulinotropic peptide (GIP) [6]. Following a meal, these incretins regulate blood glucose levels by stimulating insulin release, delaying gastrointestinal emptying, inducing satiety, decreasing glucagon release, and preserving beta cell mass [7]. As a result, inhibition of their degradation would aid in establishing glucose homeostasis from multiple perspectives, along with additional benefits of weight neutrality and a very low risk of hypoglycemia [8]. Since DPP4 rapidly inactivates incretin hormones, it is rational to assume that an increase in DPP4 activity might contribute to the impaired incretin effect observed in such DM patients [9]. However, if we study the functional molecular biology of this enzyme, we may find that this enzyme has more to offer.

The protease DPP4, which also exists as a cell surface antigen and is known as a cluster of differentiation 26 (CD26), is fundamentally a multifunctional trans-membrane glycoprotein with catalytic and signal transducing properties. It is ubiquitously displayed on various cell types, including epithelial cells, fibroblasts, and leukocyte subsets, with a dense predilection toward expression in brush border regions of the small intestine and kidney [10]. It is cleaved off the membrane into a soluble form and released into circulation by a process called shedding by the involvement of matrix metalloproteases (MMPs) [11]. Both membrane-bound DPP4 and soluble DPP4 are enzymatically active and not only inactivate peptides such as the incretins but also act upon a variety of other substrates, including post-translational modification of chemokines, neuropeptides, and different regulatory moieties [12]. Moreover, its specific protein binding properties have also been linked with immune-regulatory activities, and DPP4, as CD26, is considered to have a co-stimulatory effect on T-cell activation and proliferation [13]. Furthermore, lymphocyte-epithelial cell adhesion experiments have revealed that the adhesion of several T-cell lines is greatly dependent on CD26 [14]. In addition, Lamers et al. showed that it can act as an adipokine that may be linked to insulin resistance (IR) in an autocrine and paracrine fashion [15]. Given the multi-arrayed pleiotropic actions, DPP4 has the profound potential to affect the humoral milieu as a novel molecule contributing to the pathogenic crossways in terms of association with inflammation, T cell activation, autoimmunity, insulin resistance, and other regulatory processes. However, data regarding DPP4 activity measurements are very few, especially in the young population. A few studies on activity measurements in young people mainly focused on T1DM, and none observed the activity in newly diagnosed, untreated cases [16-19]. A molecule that lies at the intersection with implications upon these attributes demands exploration of DM in the young population. So, the study aimed to measure serum DPP4 enzyme activity in youth-onset T2DM and compare it with that in people with normal glucose tolerance (NGT).

## Materials and methods

Study design and subjects

This observational cross-sectional study measured serum DPP4 enzyme activity in young subjects between 13 and 30 years of age as, by definition, the term ‘diabetes of young’ is defined as ‘DM occurring in people with onset before the age of 30 years’ [20]. Individuals with pregnancy, a history of acute illness, pre-existing comorbidities, and drug-induced or other known causes of secondary DM were excluded. All the participants with DM had phenotypical T2DM. For comparison, the study recruited an equal number of young participants with NGT. The sample size was calculated according to the following formula: n = (Z_α_+ Z_β_)^2^ × ( σ_1_^2^ +σ_2_^2^)/(μ_1_-μ_2_)^2^, where μ_1_=mean of DPP4 activity in DM=1.29, σ_1_=SD of DPP4 activity in DM=0.38, μ_2_=mean of DPP4 activity in NGT=1.0, σ_2_= SD of DPP4 activity in NGT=0.28 [21], Zα=1.96 at 95% confidence level, and Zβ =1.28 at 90% power. Conferring to the above formula, the sample size was approximately 28 for each group.

Study procedure

Subjects were recruited by purposive sampling from the Department of Endocrinology, Bangabandhu Sheikh Mujib Medical University (BSMMU), Dhaka, on a referral basis from June 2017 to December 2018. Participants underwent a two-sample 75gm oral glucose tolerance test (OGTT) and were labeled as DM or normal glucose tolerance (NGT) group per ADA criteria [22]. When the NGT arm was filled up, screening OGTT was continued to enroll the desired number of DM only. The relevant history of each participant was obtained; waist circumference (WC), hip circumference (HC), and blood pressure (BP) were measured, whereas the waist-hip ratio (WHR) and body mass index (BMI) were calculated according to the standard procedure and recorded in the case record form. Those on blood pressure medications or with blood pressure ≥140/90 mmHg were considered to have hypertension (HTN). Fasting venous blood was collected and centrifuged, and serum was stored at -80^0^C in a freezer until laboratory analysis.

Analytic methods

Plasma glucose was analyzed using a Dimension EXL 200 Integrated Chemistry System (Siemens, Germany). To measure serum DPP4 activity, the samples were assayed at the Molecular Biotechnology Division of the National Institute of Biotechnology (NIB), Savar, Dhaka. A sandwich enzyme-linked immunosorbent assay (ELISA)-based DPPIV assay kit from Enzo Life Sciences (BML-AK498-0001) was used to measure DPP4 activity. Absorbance (OD) was read using the microplate reader model LMPR-A11, manufactured by Labtron Equipment Ltd., United Kingdom, and DPP4 activity was calculated with the equation: slope (OD/min) x conversion factor (μM/OD) x assay vol (μL) according to the kit manufacturer’s protocol expressed as pmol/min [23]. 

Ethical aspects

The study was run following approval from the Ethical Committee of the Institutional Review Board (IRB) of BSMMU (No. BSMMU/2017/6490). Informed written consent was obtained from all the participants or their guardians.

Statistical analysis

Data were analyzed using IBM SPSS Statistics for Windows, Version 24 (Released 2016; IBM Corp., Armonk, New York, United States). Results were described in frequencies or percentages for qualitative values and mean (± SD) for quantitative values with normal distribution. Subgroups (DM vs. NGT) were compared using Fisher’s exact test, the chi-square test, or an unpaired independent t-test as applicable. Multivariable linear regression was done to analyze whether the presence of DM was a determinant of DPP4 activity when adjusted for age, sex, BMI, and HTN. p-values <0.05 were considered statistically significant.

## Results

Characteristics of the study subjects

The clinical and biochemical characteristics of the study participants are presented in Table 1. Participants with DM showed significantly higher frequency of central obesity measured by WC (p=0.035), systolic blood pressure (p=0.006), diastolic blood pressure (p=0.005), and presence of acanthosis nigricans (p=0.010) than the NGT group. However, no significant difference was perceived for a positive family history of DM among 1st-degree relatives, smoking history, and WHR category.

**Table 1:** Characteristics of the study subjects (n=58) Within parentheses are percentages over the column total Significance values stand for comparison between DM and NGT groups by the independent sample t-test, chi-square test, and Fisher’s exact test as applicable. DM: diabetes mellitus; NGT: normal glucose tolerance; HC: hip circumference; BMI: body mass index; WC: waist circumference; WHR: waist-hip ratio; SBP: systolic blood pressure; DBP: diastolic blood pressure *WC: Obese>90 cm in male, >80 in female; **WHR: Obese>0.9 in male, >0.85 in female

Variables	DM	NGT	p
n	29	29	
Age (years±SD)	24.5±4.5	26.5±2.9	0.050
Gender			
Male	14 (48.3)	15 (51.7)	0.793
Female	15 (51.7)	14 (48.3)	
Family history of DM in first-degree relatives			
Present	20 (69)	15 (51.7)	0.180
Absent	9 (31)	14 (48.3)	
Smoking history			
Smoker	5 (17.2)	3 (10.3)	0.706
Non-smoker	24 (82.8)	26 (89.7)	
Acanthosis Nigricans			
Present	10 (34.5)	2 (6.9)	0.010
Absent	19 (65.5)	27 (93.1)	
*WC category			
Obese	17 (58.6)	9 (31.0)	0.035
Non-obese	12 (41.4)	20 (69.0)	
**WHR category	0.9±0.1	0.9±0.1	0.103
Obese	20 (69.0)	13 (44.8)	0.063
Non-obese	9 (31.0)	16 (55.2)	
BMI (Kg/m^2^)	24.9±4.9	23.4±3.8	0.201
SBP (mm Hg)	118.8±16.9	107.6±12.9	0.006
DBP (mm Hg)	81.2±10.8	73.6±9.0	0.005

Serum DPP4 activity of the study participants

**Figure 1:**
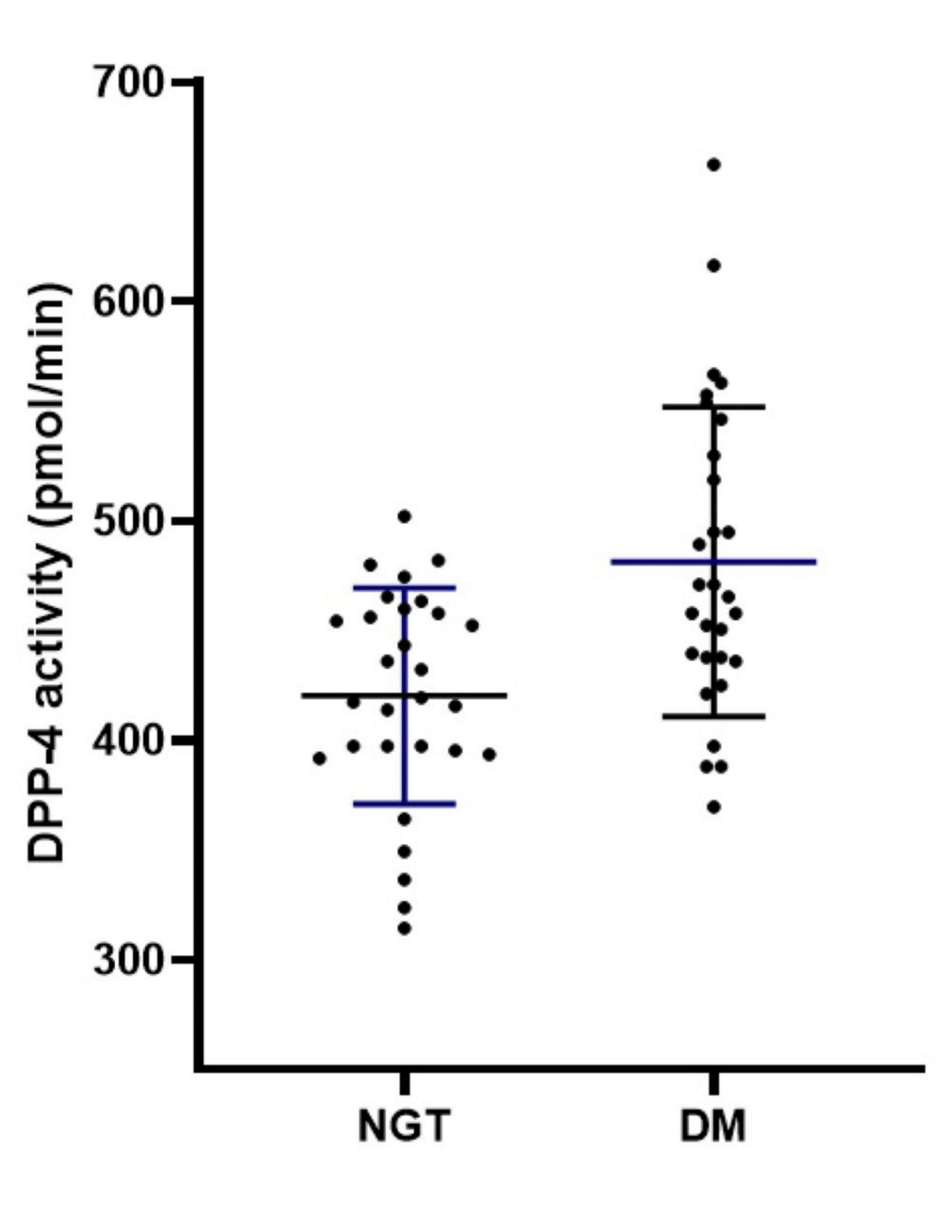
Comparison of serum DPP4 activity between study subjects with NGT (n=29) and DM (n=29) p<0.001, by the unpaired t-test; error bars represent standard deviation DPP4: dipeptidyl peptidase 4; NGT: normal glucose tolerance; DM: diabetes mellitus

The serum DPP4 activity of the study subjects was 450.9±67.6 pmol/min (n=58, mean±SD, 95% CI: 433.1 to 468.7). Participants with DM exhibited significantly higher serum DPP4 activity than controls (DM vs. NGT: 481.4±70.4 pmol/min. vs. 420.3±49.2 pmol/min, mean±SD; p<0.001, Figure 1).

Predictive associations of parameters with DPP4 activity

To assess whether the presence of diabetes is a determinant of DPP4 activity, multivariable linear regression analysis was performed, including DPP4 activity as a dependent variable and the following variables as explanatory variables: age, gender, BMI, the presence of HTN, and diabetes. The analysis demonstrated that diabetes is an independent contributing factor for DPP4 activity (unstandardized β=63.5, 95% CI 28.7-98.4, p=0.011), while other parameters are not (Table 2). Unstandardized β was reported to facilitate direct interpretation in terms of the units of the variables in the model.

**Table 2:** Multivariable regression analysis for clinical determinant(s) of serum DPP4 activity Adjusted R^2^=0.178; DPP4: dipeptidyl peptidase-4; BMI: body mass index; HTN: hypertension; DM: diabetes mellitus

Predictor	*Unstandardized β	95% CI		p
		Lower bound	Upper bound	
Age (years)	2.8	-1.6	7.1	0.214
Female gender	-7.3	-40.4	444.8	0.919
BMI (kg/m^2^)	1.9	-2.0	5.8	0.330
HTN	2.2	-40.5	44.8	0.856
DM	63.5	28.7	98.4	0.001

## Discussion

The present study compared serum DPP4 activity between the newly diagnosed young T2DM and NGT. It was observed that DPP4 activity was signiﬁcantly elevated in young T2DM in comparison to NGT. The presence of diabetes was an independent contributing factor to higher DPP4 activity.

While most studies agreed upon the increased activity of the enzyme, conflicting reports on the correlation between DPP-4 activity and glycemic control markers were found [16]. Mannucci et al. stated that DPP4 activity in T1DM patients was not significantly different from that in control subjects [24]. To the contrary, Iwabuchi et al. reported that serum DPP4 activity was higher in patients with T1DM than in controls independent of islet-cell antibody or glutamic acid decarboxylase antibody (GADA) status in harmony with the outcomes of Varga et al. [16,17]. DPP4 activity was also observed to be increased in T2DM [18]. In agreement with these findings, our study found significantly higher DPP4 activity in young phenotypical T2DM in comparison to controls after adjusting for age, sex, BMI, and hypertension, all of which may affect DPP4 activity [25,26]. Studies observed that increasing age, BMI, and female sex negatively affect the DPP4 level. There is no established direct relationship between hypertension and DPP4 activity, however, a link is suggested as there is a context-dependent impact of DPP4 inhibitors on blood pressure. The mechanism(s) underlying the increased DPP4 activity are yet to be elucidated. Several hypotheses are speculated in the context of the features of diabetes. First and foremost, the impact of chronic hyperglycemia is suggested. In experimental models, a two-week exposure to high glucose levels in human glomerular endothelial cells increased DPP4 mRNA expression and enzymatic activity [27]. It was also observed that high glucose load signiﬁcantly enhanced DPP4 mRNA expression in human hepatocyte line HepG2 cells, suggesting that DPP4 expression is directly affected by hyperglycemia [28]. Secondly, it is also reported that serum DPP4 concentration is signiﬁcantly correlated with serum advanced glycation end-products (AGEs) in T2DM patients [19]. Thus, chronic hyperglycemia and accumulated AGEs may have affected the higher DPP4 activity in our diabetes subjects than in the healthy controls, who should exhibit lower AGEs.

The cause-and-effect relationship between hyperglycemia and increased DPP4 activity is still not established. The outcome of a prospective cohort study conducted on the Chinese adult population demonstrated that increased plasma DPP4 activity predicted new-onset hyperglycemia [18]. Chronic subclinical inflammation is hypothesized to be involved in the pathogenesis of hyperglycemia [29]. Despite numerous reports of inflammatory markers that predict the development of hyperglycemia, the metabolic signaling pathways linking inflammation to hyperglycemia are less well understood. However, DPP4 exerts a catalytic activity on various peptide substrates, including chemokines and inflammatory cytokines. In addition, soluble DPP4 binds to insulin-like growth factor II/mannose 6-phosphate receptor (IGF-II/M6P-R) and is taken up by CD14 positive monocytes, increasing their antigen-presenting activity and T-cell proliferation [30]. These findings suggest that DPP4 may be involved in developing tissue inflammation in the body. Because key pathophysiological changes in hyperglycemia include chronic low-grade inflammation, it could be hypothesized that DPP4 activity may play an essential role in promoting hyperglycemia through its pro-inflammatory actions. Moreover, increased DPP4 activity enhances the degradation of GLP-1 and lipid accumulation, and these factors may also contribute to the development of hyperglycemia leading to DM. Such findings may not only suggest a pathophysiological implication but also reflect the future possibility of being an option for disease-modifying therapeutic targets in the DM of young people.

The cross-sectional nature of the study performed on a small number of subjects precludes the deduction of any causal relationship between the observed associations. Although the samples were obtained in the fasting state and DPP4 activity was reported to be not affected by meal ingestion or acute changes in plasma glucose [9], the influence of heterogeneous confounders, including the impact of adolescence, cannot be ruled out. Moreover, the population data of DPP4 activity was not available to compare with a national reference value. Indeed, further longitudinal studies are required to confirm this enzyme's significance in the pathogenic context of young-onset DM. Since population data about the measured activity are lacking, large-scale DM type-specific studies are warranted to elucidate the question of whether strong pathogenic implications of DPP4 activity in various types of DM exist or not. Community-based large-scale studies in the Bangladeshi young population should be conducted to evaluate the role of the DPP-4 enzyme as a prospective circulatory determinant of β cell dysfunction leading to new-onset hyperglycemia. Furthermore, long-term prospective studies with periodic measurement of the enzyme’s activity and glycemic status with its consequences may provide a valuable perception for developing future disease-modifying therapeutics in the DM of young people.

## Conclusions

DPP4 activity was signiﬁcantly elevated in newly diagnosed young T2DM. In addition, the presence of diabetes was an independent contributing factor for higher DPP4 activity. These data suggest a potential role for DPP4 in diabetes in the young population and provide a valuable concept for developing future therapies and treatment recommendations for the disease. There may be a pathophysiological implication of DPP4 activity in youth-onset DM as it has impact on both inflammatory cascades and incretin-based pathways of glucose regulation. However, it is unclear whether elevated DPP4 activity is the cause or the effect of T2DM in young people. Further study investigation in a larger sample is warranted to elucidate the question of whether a strong association between DPP4 activity, glycemia, and insulin sensitivity exists or not.

## References

[1] Sun H, Saeedi P, Karuranga S (2022). IDF Diabetes Atlas: global, regional and country-level diabetes prevalence estimates for 2021 and projections for 2045. Diabetes Res Clin Pract.

[2] Chan JC, Malik V, Jia W, Kadowaki T, Yajnik CS, Yoon KH, Hu FB (2009). Diabetes in Asia: epidemiology, risk factors, and pathophysiology. JAMA.

[3] Hughes JW, Bao YK, Salam M (2019). Late-onset T1DM and older age predict risk of additional autoimmune disease. Diabetes Care.

[4] Candler TP, Mahmoud O, Lynn RM, Majbar AA, Barrett TG, Shield JP (2018). Continuing rise of type 2 diabetes incidence in children and young people in the UK. Diabet Med.

[5] Tsalamandris S, Antonopoulos AS, Oikonomou E (2019). The role of inflammation in diabetes: current concepts and future perspectives. Eur Cardiol.

[6] Omar B, Ahrén B (2014). Pleiotropic mechanisms for the glucose-lowering action of DPP-4 inhibitors. Diabetes.

[7] Cornell S (2012). Differentiating among incretin therapies: a multiple-target approach to type 2 diabetes. J Clin Pharm Ther.

[8] Maruthur NM, Tseng E, Hutfless S (2016). Diabetes medications as monotherapy or metformin-based combination therapy for type 2 diabetes: a systematic review and meta-analysis. Ann Intern Med.

[9] Ryskjaer J, Deacon CF, Carr RD, Krarup T, Madsbad S, Holst J, Vilsbøll T (2006). Plasma dipeptidyl peptidase-IV activity in patients with type-2 diabetes mellitus correlates positively with HbAlc levels, but is not acutely affected by food intake. Eur J Endocrinol.

[10] Augustyns K, Bal G, Thonus G (1999). The unique properties of dipeptidyl-peptidase IV (DPP IV / CD26) and the therapeutic potential of DPP IV inhibitors. Curr Med Chem.

[11] Röhrborn D, Eckel J, Sell H (2014). Shedding of dipeptidyl peptidase 4 is mediated by metalloproteases and up-regulated by hypoxia in human adipocytes and smooth muscle cells. FEBS Lett.

[12] Zhong J, Rao X, Rajagopalan S (2013). An emerging role of dipeptidyl peptidase 4 (DPP4) beyond glucose control: potential implications in cardiovascular disease. Atherosclerosis.

[13] Iwata S, Yamaguchi N, Munakata Y (1999). CD26/dipeptidyl peptidase IV differentially regulates the chemotaxis of T cells and monocytes toward RANTES: possible mechanism for the switch from innate to acquired immune response. Int Immunol.

[14] Ginés S, Mariño M, Mallol J (2002). Regulation of epithelial and lymphocyte cell adhesion by adenosine deaminase-CD26 interaction. Biochem J.

[15] Lamers D, Famulla S, Wronkowitz N (2011). Dipeptidyl peptidase 4 is a novel adipokine potentially linking obesity to the metabolic syndrome. Diabetes.

[16] Varga T, Somogyi A, Barna G (2011). Higher serum DPP-4 enzyme activity and decreased lymphocyte CD26 expression in type 1 diabetes. Pathol Oncol Res.

[17] Iwabuchi A, Kamoda T, Saito M, Nozue H, Izumi I, Hirano T, Sumazaki R (2013). Serum dipeptidyl peptidase 4 activity in children with type 1 diabetes mellitus. J Pediatr Endocrinol Metab.

[18] Zheng T, Gao Y, Baskota A, Chen T, Ran X, Tian H (2014). Increased plasma DPP4 activity is predictive of prediabetes and type 2 diabetes onset in Chinese over a four-year period: result from the China National Diabetes and Metabolic Disorders Study. J Clin Endocrinol Metab.

[19] Tahara N, Yamagishi S, Takeuchi M (2013). Serum levels of advanced glycation end products (AGEs) are independently correlated with circulating levels of dipeptidyl peptidase-4 (DPP-4) in humans. Clin Biochem.

[20] Das AK (2009). Type 2 diabetes mellitus in the young. Clinical Endocrinology and Diabetes Mellitus: A Comprehensive Text.

[21] Osawa S, Kawamori D, Katakami N (2016). Significant elevation of serum dipeptidyl peptidase-4 activity in young-adult type 1 diabetes. Diabetes Res Clin Pract.

[22] (2017). 2. Classification and diagnosis of diabetes. Diabetes Care.

[23] (2017). Enzo life sciences. DPPIV/CD26 Assay Kit for Biological Samples Designed to measure protease activity of DPPIV/CD26. Retrieved 12. http://www.enzolifesciences.com/BML-AK498/dppiv-cd26-assay-kit-for-biological-samples/.

[24] Mannucci E, Pala L, Ciani S (2005). Hyperglycaemia increases dipeptidyl peptidase IV activity in diabetes mellitus. Diabetologia.

[25] Stenlid R, Manell H, Halldin M (2018). High DPP-4 concentrations in adolescents are associated with low intact GLP-1. J Clin Endocrinol Metab.

[26] Jackson EK, Mi Z, Tofovic SP, Gillespie DG (2015). Effect of dipeptidyl peptidase 4 inhibition on arterial blood pressure is context dependent. Hypertension.

[27] Pala L, Mannucci E, Pezzatini A (2003). Dipeptidyl peptidase-IV expression and activity in human glomerular endothelial cells. Biochem Biophys Res Commun.

[28] Miyazaki M, Kato M, Tanaka K (2012). Increased hepatic expression of dipeptidyl peptidase-4 in non-alcoholic fatty liver disease and its association with insulin resistance and glucose metabolism. Mol Med Rep.

[29] Pickup JC (2004). Inflammation and activated innate immunity in the pathogenesis of type 2 diabetes. Diabetes Care.

[30] Ikushima H, Munakata Y, Iwata S, Ohnuma K, Kobayashi S, Dang NH, Morimoto C (2002). Soluble CD26/dipeptidyl peptidase IV enhances transendothelial migration via its interaction with mannose 6-phosphate/insulin-like growth factor II receptor. Cell Immunol.

